# Shaping Ability of Reciproc, UnicOne, and Protaper Universal in Simulated Root Canals

**DOI:** 10.1155/2015/690854

**Published:** 2015-04-09

**Authors:** Etevaldo Matos Maia Filho, Cláudia de Castro Rizzi, Matheus Bandeca Coelho, Sara Freitas Santos, Luzia Mayanne Oliveira Costa, Ceci Nunes Carvalho, Rudys Rodolfo de Jesus Tavarez, Janir Alves Soares

**Affiliations:** ^1^University Ceuma, Rua Josué Montello No. 1, Renascença II, 65075-120 São Luís, MA, Brazil; ^2^Universidade Federal dos Vales do Jequitinhonha e Mucuri, Rua da Glória, 187 Centro, 39100-000 Diamantina, MG, Brazil

## Abstract

The study aimed to compare the shaping effects, preservation of the original curvature, and transportation of the apical foramen of Reciproc (VDW, Munich, Germany), UnicOne (Medin, Nové Město na Moravě, Czech Republic), and Protaper Universal (Dentsply Maillefer, Ballaigues, Switzerland) in simulated root canals. Thirty resin blocks with simulated curved root canals were distributed into three groups (*n* = 10), and prepared using Reciproc (RCp), UnicOne (UnO) and the Protaper Universal (PTu). Standardized photographs were taken before and after the instrumentation, after which they were superimposed. Measurements were taken of the quantity of resin removed from the inner and outer walls of the curvature at 6 levels, the curvature angles before and after instrumentation, and the transportation of the apical foramen. RCp obtained the highest values for amount of resin removed from the inner wall while UnO demonstrated similar shaping on both the inner and outer walls. PTu produced the greatest transportation of foramen when compared to the reciprocating instruments. There was no significant difference between the groups in terms of the change in angle (*P* > 0.05). All the instruments were capable of maintaining the original curvature of the root canal; however, the UnO, which used reciprocating movement, produced more conservative shapes with lower foramen transportation.

## 1. Introduction

The main objective of endodontic treatment is to clean the root canal, giving it a conical shape in the direction of crown to apex, preserving the original curvature; however, during instrumentation divergences from its original shape may occur at some point [1]. These changes may have a negative impact on the quality of the filling of the root canals [2]. Moreover, at the present time there are no instruments capable of completely cleaning the root canal system [3, 4], particularly the apical region, without producing undesirable consequences [5, 6].

Since the introduction of nickel-titanium instruments in 1988 by Walia et al. [7], the use of rotary instruments has revolutionized endodontic treatment, reducing operator fatigue and treatment time and minimizing errors associated with the use of stainless steel instruments [8].

In 2008, Yared proposed a new instrumentation technique using a single instrument with reciprocating movement [9]. This new technique presented some advantages such as a reduction of the number of instruments and, consequently, the reduction of the cost and the operator fatigue, as well as the elimination of possible cross-contamination during treatment. In addition, it has been proven to increase the longevity of the instrument when compared to others that use rotary movement [10].

In the kinematics of the reciprocating movement, the instrument rotates in both an anticlockwise and clockwise direction with a difference of 120° between the two movements. For every three cycles, there is one complete rotation of the instrument. In this way, 10 cycles of alternating movement are performed every second, equivalent to 300 rpm. When the instrument rotates in the direction of the cut, it moves forward and attaches itself to the dentin in order to cut it. When it rotates in the opposite direction, the instrument immediately disengages. The final outcome is the instrument advancing into the root canal with just a light apical pressure. This action reduces cyclic fatigue [9–11] and requires shorter working time [12].

At the present time, different reciprocating, single-use instruments have been introduced in the market. Reciproc file (VDW, Munich, Germany) has been shown to be safe and effective in the preparation of curved root canals [1, 13]. The new file (UnicOne, Medin, Nové Město na Moravě, Czech Republic) has an inactive tip and a variable, triangular cross-section design; however, studies are required to evaluate this new instrument's ability to preserve the root canal anatomy and its effect on the transportation of foramen. The objective of this study, therefore, was to evaluate the shaping ability and preservation of the original canal curvature, as well as the transportation of the apical foramen using the UnicOne instrument, and to compare UnicOne with a reciprocating instrument widely acclaimed in the literature (Reciproc) and with a rotary technique (Protaper Universal System, Dentsply Maillefer, Ballaigues, Switzerland).

## 2. Material and Methods

### 2.1. Resin Blocks

Thirty transparent resin blocks were used with simulated, standardized (ISO 15, taper 0.02) curved root canals (IM do Brasil Ltda., São Paulo, SP, Brazil), with a length of 19 mm of which 13 mm represents the straight, coronal part and 6 mm the curved apical portion, with an angle of curvature of 35° [14]. The blocks were divided into three groups (*n* = 10) and prepared using Reciproc (RCp), UnicOne (UnO), and the Protaper Universal (PTu).

### 2.2. Instrumentation of the Root Canals

The working length (WL) was determined when the tip of the #10 file (Dentsply/Maillefer, Ballaigues, Switzerland) was visualized at the canal terminus, and the file was withdrawn 1 mm.

All the canals were instrumented by the same operator using the Gold Reciproc motor (VDW, Munich, Germany) with 25 mm single-use files. RCp (R25-25/0.08) and UnO (25/0.06) files were used by employing reciprocating movements while the PTu files (SX, S1, S2, F1, and F2) were used by applying rotary movement (speed of 300 rpm and torque of 320 gcm) using 3 pecking movements until the WL was reached. The canals were irrigated with distilled water using syringe and needle (Endo-Eze Irrigator 27G; Ultradent, USA). The instrumentation was finished when the instrument reached the WL.

### 2.3. Evaluation of Canal Preparation

Photographs were taken before and after the instrumentation of the simulated root canals using a digital camera (EOS Rebel T3i, Canon, Japan) attached to a copy stand (Tokina Company Ltd., Hong Kong, China). The resulting TIFF images were processed digitally using Adobe Photoshop CS6 (Adobe System Incorporated, San Jose, California, USA) in order to demarcate the area of the canal. The canals were demarcated by way of the* Quick Selection* tool and the* Paint Bucket* tool used to fill in the canals in white (image before instrumentation) and in black (image after instrumentation).

**Figure 1:**
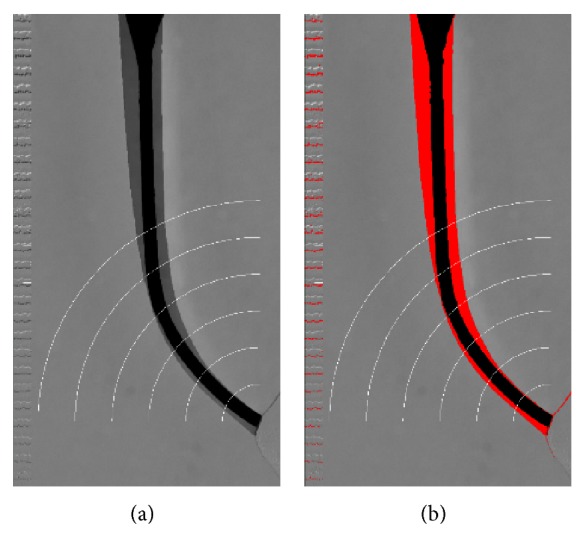
Images extracted from the canals before and after instrumentation and with the demarcation of the six apical levels (a) and after detachment for the calculation of the areas of resin removed (b).

The images of the simulated root canals before and after instrumentation were superimposed using program Regeemy version 0.2.43 (http://wiki.dpi.inpe.br/doku.php?id=wiki:regeemy). The part corresponding to the canal's curved region (6 mm) was divided into six levels. The basis for this division was a point in the center of the canal at the tip of the apex in the preinstrumentation image. From this point, six arcs are described with radii at intervals of 1 mm (AutoCad 2014, Autodesk Inc., San Rafael, CA, USA) (Figure 1(a)). The final image was analyzed using the application ImageJ 1.48r (http://rsbweb.nih.gov/ij/) and calibrated with the use of the* Analyze/Set Scale* tool with the aid of a millimeter ruler, photographed together with the resin block. The* Adjust/Threshold* tool was used to highlight the prepared region of the root canal (Figure 1(b)) and, with the aid of the* ROI Manager *and* Wand* tools, the internal and external areas of the curvature were calculated at each level.

The transportation of the simulated apical foramen was calculated by measuring (in millimeters) the thickness of resin removed by the instrumentation in the most apical portion of the root canal turned towards the outside of the curvature.

The angles of the canals before and after instrumentation were calculated using the ImageJ* Tool Angle* tool, in accordance with the Schneider method [14], by two independent evaluators. The mean values were used. The difference between the values of the angles of curvature at the start and after instrumentation served as the parameter for evaluating the maintenance of the original curvature of the canals.

### 2.4. Statistical Analysis

The mean and standard deviation of the worn areas were calculated for each group at each level. Once the data had been submitted for normality analysis, the variance analysis test for a single factor (one-way ANOVA) was employed to assess whether a significant difference existed in the amount of resin removed from inner and outer areas of the curvature between the groups and similarly for the transportation of the foramen and change in angle. The post hoc Tukey test was used when a significant difference between the groups existed. The statistical program used was the SPSS 21.0 (IBM, Armonk, NY, USA) with a level of significance of 5%.

## 3. Results

**Figure 2:**
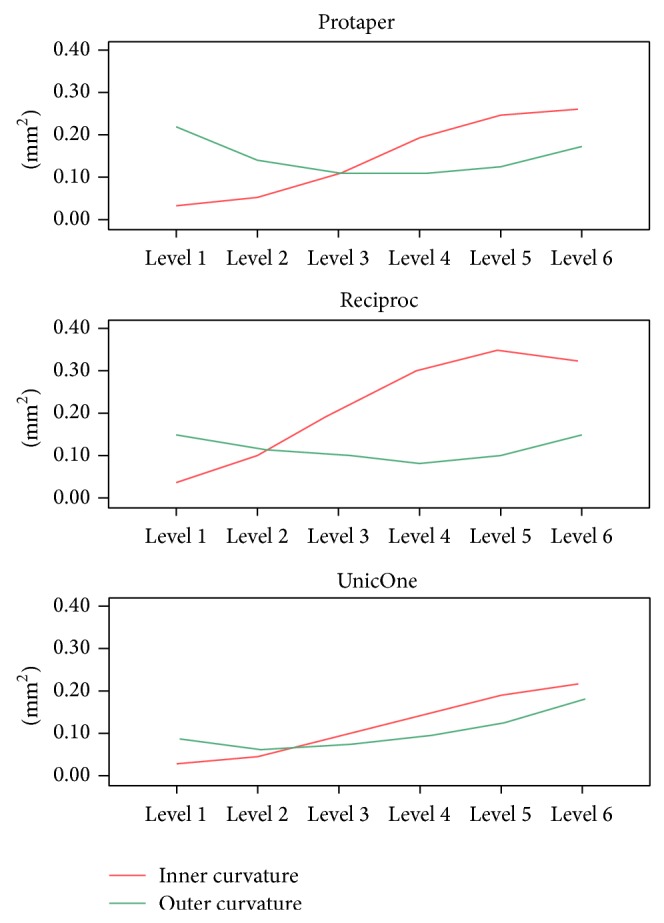
Mean of the areas of resin removed, in square millimeters, of the inner and outer curvatures amongst the instruments evaluated at the six apical levels.

The mean and standard deviation of the amount of resin removed, for each level, are presented in Table 1. There was a significant difference between the groups for the resin removed from both the internal (*P* = 0.001) and external (*P* = 0.006) areas of the curvature. The RCp instrument produced the largest amount of resin removal at all evaluated levels on the internal part of the curvature, followed by the PTu and UnO. As for the PTu, it produced a higher amount of resin removal at levels 1 to 4 of the external part of the curvature, in other words, the levels closest to the canal terminus (Figure 2).

Table 2 shows the values for the proportion of resin removal between the inner/outer walls of the curvature. The highest values were obtained with the RCp at levels 3 to 6 while at levels 1 and 2, the PTu system attained the lowest values.

As regards the transportation of the simulated apical foramen, the highest values were found in group 3 (PTu), followed by group 1 (RCp) and group 2 (UnO). There was a significant difference between groups 3 and 2 (*P* = 0.014) (Table 3).

Concordance between examiners with the measurement of the angles of root canal curvature was arrived at using the intraclass correlation coefficient (ICC), which showed agreement of 0.934 (*P* = 0.001) for measurements taken prior to instrumentation and 0.953 (*P* = 0.001) for those taken after instrumentation.

The means (± standard deviation) of the difference between the angle before and after instrumentation are shown in Table 3. There was no significant difference in the change of angle between the groups (*P* = 0.385).

## 4. Discussion

The purpose of this study was to compare the shaping ability of three instruments recommended for the preparation of curved canals. The following parameters were evaluated: amount of resin removed from the inner and outer parts of the root canal curvature at six levels, the transportation of the simulated apical foramen, and the maintenance of the original curvature of the simulated curved root canals.

Several methods can be used to evaluate and compare root canal preparations before and after instrumentation [2, 13, 15]; however, the use of simulated root canals in resin blocks enables the standardization of the canal morphology such as the angle, curvature radius, diameter, and length of the root canal. This method, however, has the disadvantage of being unable to evaluate the root canal and its cross-section in a three-dimensional view. Moreover, the mechanical properties of the resin are different from those of human teeth. However, as the conditions are identical for the different instruments, the results obtained using simulated root canals in resin blocks may be validated for natural teeth [16, 17].

In this study, the finalization of the preparations was always carried out using instruments with a tip diameter equivalent to size 25; however the tapers were not congruent. While the UnO has a constant taper of 0.06, the RCp, R25, and PTu, in the initial three millimeters, have a taper of 0.08. There is a reduction of 4.3% in the remainder with the R25 while, for the Protaper Universal F2, from D4 to D8, the taper reduces progressively as far as 0.04 and, at the end, in the direction of D16, it is reduced to 0.03.

The ideal instrumentation should follow the anatomy of the root canal; that is, the dentin removed on the inner and outer wall of the root canal should maintain the same proportion and cause less displacement of the apical foramen [18]. In this regard, the three techniques caused similar shaping of the inner and outer regions of the curvature (Figure 1), a higher resin removal from the inner region of the curvature at levels 3 to 6 (coronal level) and greater resin removal at levels 1 and 2 of the outer portion of the curvature (apical level). This finding shows that all the instruments possessed a tendency to straighten the root canal; however it was the UnO that maintained the best proportion of shaping between the inner/outer walls along the whole length of the canal (values closest to 1).

The PTu sequence produced the highest resin removal from the outer part of the curvature at the four levels closest to the apex and was also the one which produced the largest transportation of the simulated apical foramen. These undesirable effects possibly occurred due to the greater number of instruments used or could be associated with rotary movement, which has been proved to be less effective in maintaining the original curvature of root canals when compared with instruments employing reciprocating movement [2, 19, 20].

The transportation of foramen has a negative impact on the effectiveness of the seal provided by the filling [21]. In this regard, the UnO caused less displacement compared to the other instruments and, in addition, was the instrument which maintained the best proportion of resin removal between the inner and outer walls of the curvature. This probably occurred due to the lower metallic mass of this instrument and the smaller taper, which interferes with flexibility and could have provided a better adaptation to the canal walls during instrumentation.

Under the study conditions, both the transportation of foramen and the resin removed from the outer wall of the root canals were greater when using the PTu sequence, showing a greater propensity for these instruments to straighten the original shape of the canal during instrumentation. This finding showed that the instruments that were used with reciprocating movement (RCp and UnO) preserved the outer part of the curvature more than those instruments that used rotary movement. However, there is need for further studies comparing other makes of instrument in order to confirm this assertion.

The instruments tested did not produce a significant difference in the modification of the root canal curvature. Similar results were observed by other authors [19, 22] and agree with Bürklein et al. [13] who also found no difference between the use of rotary and reciprocating instruments.

## 5. Conclusions

Within the limits of this simulated* in vitro* study, all the instruments were capable of maintaining the original curvature of the root canal; however, the UnicOne, which used reciprocating movement, produced more conservative shapes with lower foramen transportation.

## Figures and Tables

**Table 1:** Mean ± standard deviation (mm^2^) of resin removed from the internal/external areas at all levels evaluated.

	Groups	Level 1	Level 2	Level 3	Level 4	Level 5	Level 6	Total
Internal area	PTu^A^	0.0321 (±0.0141)	0.0514 (±0.0276)	0.1057 (±0.0341)	0.1930 (±0.0388)	0.2468 (±0.0698)	0.2592 (±0.0928)	0.1479 (±0.1044)
	RCp^B^	0.0402 (±0.0163)	0.1061 (±0.0525)	0.2117 (±0.0584)	0.3083 (±0.0799)	0.3535 (±0.0717)	0.3277 (±0.0792)	0.2246 (±0.1329)
	UnO^C^	0.0285 (±0.0192)	0.0456 (±0.0187)	0.0909 (±0.0244)	0.1413 (±0.0441)	0.1894 (±0.0470)	0.2171 (±0.0489)	0.1188 (±0.0786)

External area	PTu^A^	0.2166 (±0.0858)	0.1409 (±0.0398)	0.1086 (±0.0403)	0.1072 (±0.0440)	0.1246 (±0.0476)	0.1705 (±0.0523)	0.1447 (±0.0647)
	RCp^AB^	0.1540 (±0.0927)	0.1223 (±0.0890)	0.1075 (±0.0719)	0.0852 (±0.0482)	0.1019 (±0.0668)	0.1545 (±0.0828)	0.1203 (±0.0777)
	UnO^B^	0.0845 (±0.0535)	0.0616 (±0.0424)	0.0744 (±0.0544)	0.0933 (±0.0514)	0.1252 (±0.0607)	0.1817 (±0.0644)	0.1035 (±0.0663)

Significant difference between the groups (one-way ANOVA). Different letters: significant difference *P* < 0.05 (Tukey test).

**Table 2:** Proportion of resin removed from the internal/external part of the curvature of the different groups for each level evaluated.

	Groups	Level 1	Level 2	Level 3	Level 4	Level 5	Level 6
Proportion, internal/external	PTu	0.15	0.36	0.97	1.80	1.98	1.52
	RCp	0.26	0.87	1.97	3.62	3.47	2.12
	UnO	0.34	0.74	1.22	1.51	1.51	1.19

**Table 3:** Mean values with their respective values for standard deviation, transportation of foramen (mm), and change in angle (degrees) between the groups evaluated.

	Groups	Mean (± standard deviation)	*P*
Transportation of foramen	PTu^A^	0.219 mm (±0.090 mm)	0.019^*^
	RCp^AB^	0.156 mm (±0.120 mm)	
	UnO^B^	0.092 mm (±0.056 mm)	

Change in angle	PTu	1.09° (±2.42°)	0.385
	RCp	0.60° (±2.09°)	
	UnO	−0.31° (±2.30°)	

^*^Significant difference between the groups (one-way ANOVA).

Different letters: significant difference *P* < 0.05 (Tukey test).
